# The mutual relationship of the policymakers, providers, and the community on the children’s oral health; New windows for more discussions

**DOI:** 10.1186/s13690-023-01073-8

**Published:** 2023-04-25

**Authors:** Peivand Bastani, Abdosaleh Jafari, Diep Hong Ha

**Affiliations:** 1 School of Dentistry, UQ Oral Health Centre, The University of Queensland, Brisbane, QLD 4006 Australia; 2 College of Health and Human Sciences, Charles Darwin University, Alice Springs, NT 0870 Australia; 3 Health Human Resources Research Center, School of Health Management and Information Sciences, Shiraz University of Medical Sciences, Almas Build. 29 valley Ghasr dasht St. PO.COD: 71336-54361, Shiraz, Iran

**Keywords:** Children oral health, Oral health policymaking, Microsystem, Mesosystem, Exosystem, Macrosystem, And chronosystem

## Abstract

**Background:**

The multidisciplinary and comprehensive nature of children`s oral health with mutual interactions among various determinants makes the area a window of more discussion among oral health policymakers, stakeholders, providers, and other interested parties. This commentary presents a triangle framework of the children`s oral health, including all the above groups, for new discussions in oral health policymaking.

**Main body:**

Three leading influencers could be recognised in children`s oral health as a triangle despite the contextual differences among the countries. The first angle, *Families and community*, determine the individual background, demographic, biological, genetic, and psychological factors, as well as community-based and social background, including cultural and socioeconomic factors. The second angle, *Oral health providers*, includes a variety of determinants from the provider`s perception toward oral health provision of services to availability of dental services, teledentistry and digital technology, surveillance, and monitoring systems for children`s oral health. And finally, as the third angle, *Oral health policymakers* affect the mechanism for funding dental care and supporting schemes, affordability of oral health services, regulations and standards and public education. Macro environmental policies related to the children`s ecosystem, community water fluoridation, and social marketing for promoting probiotics products` consumption are categorized in this category.

**Conclusion:**

The triangle framework of children`s oral health presents a big picture of the oral health concept at the multilevel. Although these determinant factors interact with each other, each can have a cumulative effect on children`s oral health; policymakers could try to consider them as a big picture with a systematic approach for better achievement of oral health among children considering the local and national contextual factors of the community.

**Supplementary Information:**

The online version contains supplementary material available at 10.1186/s13690-023-01073-8.

## Background

Children`s oral health is a complex multidisciplinary concept affecting their general health and is determined by many interrelated factors. There is a close relationship between determinants of physical health and subjective perceptions of health. At the same time, oral and general health share potential causes and risk factors [1].

Dental caries is considered as the major oral health problems affecting up to 60–90% of children and most adults worldwide [2]. Dental caries not only remains the biggest problem but is also determined by a multitude of factors at different levels surrounding the child, including the child’s individual, his/her family, and the community where she/he lives [3]. Evidence shows that some environmental factors, such as the children`s exposure to fluoridation along with behavioural and cultural determinants like sugar consumption and diets, particularly those with high probiotics servings, are constantly associated with dental caries prevalence and experience among children. The reason for the dental visit as a socioeconomic indicator also acts the same [4].

The utilisation of dental services is affected by many other factors such as the socioeconomic status of the household, demographic variables, cultural and behavioural factors, level of satisfaction with dental services, and the financial and beneficial supports the same as dental schemes or insurance coverage [5].

All this evidence emphasises the complex multidisciplinary nature of children`s oral health with mutual interactions among various determinants due to the complexity of the concept and multilateral interactions among the determinant factors that affect the children`s oral health at different levels. This commentary is presented a triangle framework of children`s oral health for new discussions in oral health policymaking.

## Main text

There are many recognized social determinants affecting the population`s oral health. Those significant determinants include health disparities, dental access, oral health literacy, oral health and general health, culture and oral health behaviours are notable [6]. According to the results of a systematic review, factors related to children`s characteristics, their family background, and some cultural and behavioural factors like the status of their oral hygiene, infant feeding, eating habits and brushing behaviours, along with other socio-behavioural and socioeconomic determinants at macro level are among the top factors affecting dental caries among children and their oral health [7]. At the same time, evidence revealed that lower income was associated with poor oral health. Dental access, water fluoridation and sugar consumption are among other factors that led to the significance of community and individual levels attributes [4]. Results of a scoping review have mentioned three categories of elements at the micro-individual level, meso organisational level and macro level that mainly can affect the access and utilisation of dental services [8]. Personal characteristics, health status, needs and behaviours were classified at the micro level, while social, economic, cultural, and environmental factors were among the macro level determinants. The dental health provider function, policies, practice, and insurance support were discussed at the meso organisational level [9].

**Fig. 1:**
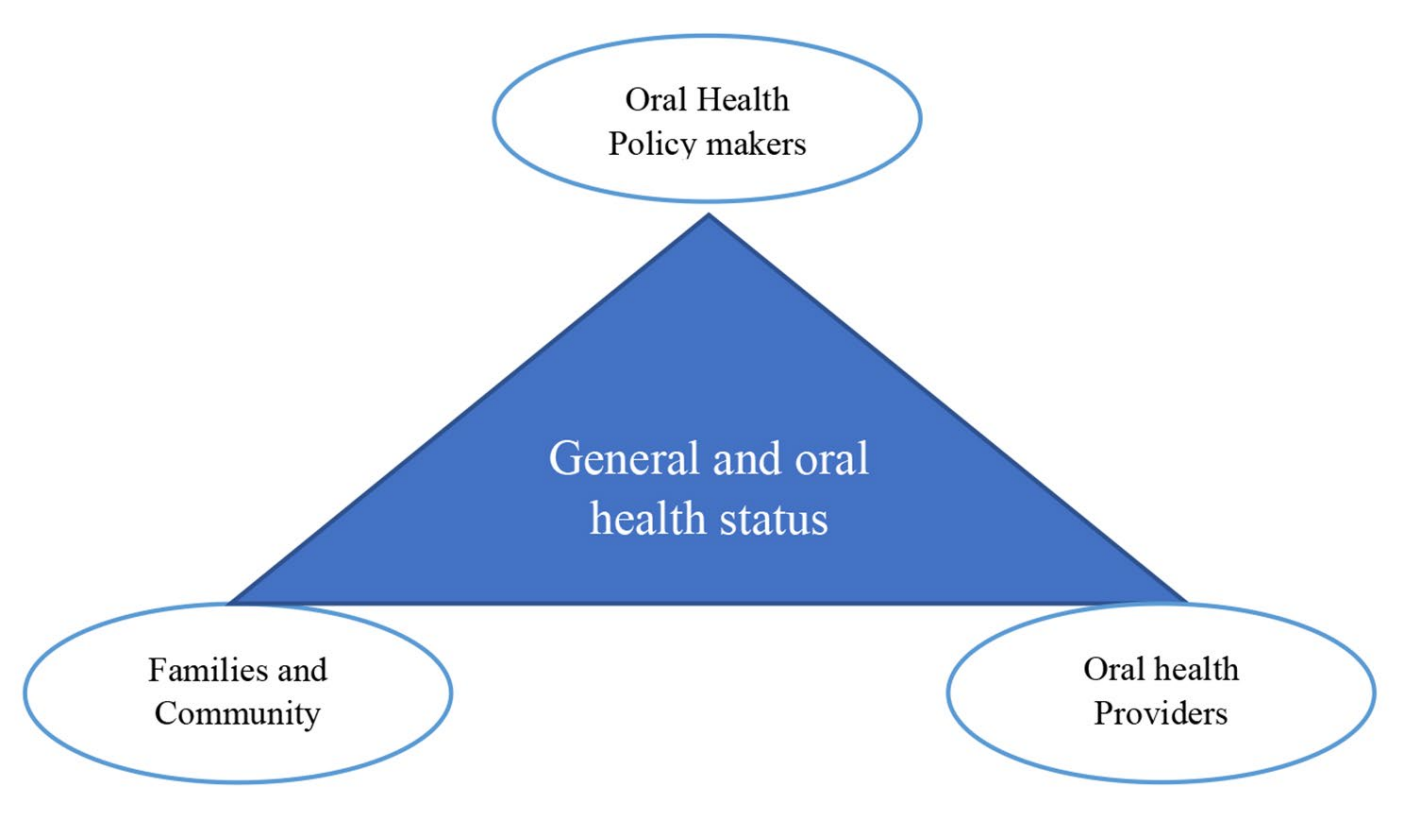
Determinant factors of children`s oral health

Considering all the above and regarding the Bronfenbrenner ecological system theory [10], which supposed five levels of microsystem, mesosystem, exosystem, macrosystem, and chronosystem surrounding a child, and combining this theory with the triangle of health system governance and policymaking which indicate three dimensions of providers, policymakers, and people [11], we can discuss different determinant factors on children oral health as follows (Fig. 1). The reason for applying the triangle of health system governance is the decisive role of policymakers, people, and healthcare providers in providing health services and evaluating health outcomes. In other words, these parties, formally and informally, make the change, monitor, and enforce those rules which obviously influence their actions, decisions, and relations and, as a result change the health policies and practices [12].

### Families and community

Many factors can be considered at this level; some of them have an individual background, such as demographic factors, biological and genetic factors, as well as psychological ones, while some others have the more community-based and social background, including cultural and socioeconomic factors (type of residence, racism, household income, neighbourhood characteristics), as well as religious factors. Two other categories of elements can be mentioned here as a mutual interaction between the children, family and the community regarding oral health behaviours and oral health literacy.

### Oral health providers

In this category, we can mention the variety of determinants from the provider`s perception toward oral health provision of services to availability of dental services (ratio of dentists and allied oral health workers), teledentistry and digital technology as well as surveillance and monitoring systems for the children`s oral health.

### Oral health policymakers

Public policies that can affect the affordability of oral health services, along with regulations and standards on food advertisement and labelling and public education, including the mechanism for funding dental care and supporting schemes such as dental insurance, are the first determinant factors in this category. In contrast, national policies such as political regimens and environmental policies related to the children`s ecosystem and community water fluoridation are considered as the second category of policymakers` perspective. Incorporating probiotics utilisation is also should be mentioned in the oral health policies and health promotion behaviours for moderating the community lifestyle.

The present commentary discussed a new viewpoint in categorising the determinant factors affecting children`s oral health based on their ecological system, from the closes system to a child; microsystem to the farthest one; the chronosystem. At the same time, the commentary makes a link via the child levels of the ecological system and the triangle of the people, healthcare providers and policymakers as the main elements of health system governance. This new approach makes this commentary different from the available knowledge and existing models, the same Fisher-Owens (2007), which mainly focused on oral health outcomes from multilevel influences of individual, family, and community [13].

## Conclusion

Children`s oral health is related to their general health and is affected by a complex and multilateral interaction of factors at three levels family/community, oral health providers and policymakers. The triangle framework of children`s oral health presents a big picture of the oral health concept at the multilevel. The national and public policies, along with the oral health guidelines and policies, can be considered at both the chronosystem and macrosystem of a child regarding the level and significance of the policy. Then this determinant factor at the top of the triangle can affect the other determinants of family/community and oral health providers. The interactions among the children, their parents and families and the surrounding community, with an emphasis on the school environment and the oral health care providers, can be flowed in all three micro, meso and exosystem surrounding a child. Although these determinant factors have their interactions with each other and each can have a cumulative effect on children`s oral health, policymakers could try to consider them as a big picture with a systematic approach for better achievement of oral health among children considering the local and national contextual factors of the community.

## Electronic supplementary material

Below is the link to the electronic supplementary material.

Supplementary Material 1

## Data Availability

All the data supporting the conclusions of this study are included within the article.
