# Supplementary material for: The mutual relationship of the policymakers, providers, and the community on the children’s oral health; New windows for more discussions
Source: Arch Public Health. 2023 Apr 25;81:69. doi: 10.1186/s13690-023-01073-8 (PMC10126996; doi:10.1186/s13690-023-01073-8)
Supplement: Supplementary file 1 — Supplementary Material 1 [file 13690_2023_1073_MOESM1_ESM.docx]

To the best of our knowledge, children oral health has a multidisciplinary and comprehensive nature with mutual interactions among various determinants. In such a context, a socioecological perspective with a complex systems approach can help oral health policymaker, stakeholders, oral health providers and other interest patties to move towards valid and robust progress and practical solutions for better child oral health globally.
This commentary contributes to the available knowledge and help to add the values in two different aspects. First, children oral health is affected by the simultaneous interactions among three levels of family/community, oral health providers and oral health policy makers. Second, this interactive triangle presents a big picture of the oral health concept at the multilevel including micro, meso, exo, macro and chrono systems. While the national public policies and the oral health guidelines can be considered at both chronosystem and macrosystem of a child, the interactions among the children, their parents and families and the surrounding community with an emphasis on the school environment and the oral health care providers can be flowed in all three micro, meso and exosystem surrounding a child.

The implications for oral health policymakers and oral health providers can be as follows:

Children oral health should not be considered as a unique phenomenon. In contrast, oral health policymakers should seek systematic approaches for better achievement of oral health among children considering the local and national contextual factors of the community considering the multilevel approach. At the same time, the interactions among the child and his/her surrounding should be highlighted to improve the whole families and the public community but not only the children to improve their oral health literacy and behaviours and consequently increase the general oral health.
